# Association Mapping Analysis of Morphological Characteristics in F_2_ Population of *Perilla* (*Perilla frutescens* L.) Using SSR Markers

**DOI:** 10.3390/plants14172799

**Published:** 2025-09-06

**Authors:** Tae Hyeon Heo, Hyeon Park, Jungeun Cho, Da Hyeon Lee, Ju Kyong Lee

**Affiliations:** 1 Department of Applied Plant Sciences, College of Agriculture and Life Sciences, Kangwon National University, Chuncheon 24341, Republic of Korea; taehyun@kangwon.ac.kr (T.H.H.); hyeonpark@kangwon.ac.kr (H.P.); jjejje2000@kangwon.ac.kr (J.C.); dahyeon@kangwon.ac.kr (D.H.L.); 2 Interdisciplinary Program in Smart Agriculture, Kangwon National University, Chuncheon 24341, Republic of Korea

**Keywords:** *Perilla frutescens*, quantitative traits, qualitative traits, marker-assisted selection, *Perilla* breeding program

## Abstract

To identify SSR markers associated with both quantitative and qualitative traits in *Perilla*, we analyzed a total of 68 individuals from an F_2_ population derived from a cross between WPC06-339 (weedy var. *crispa*) and WPF17-049 (weedy var. *frutescens*) using 40 SSR primer sets. The genetic diversity of these markers ranged from 0.464 to 0.676, with a mean value of 0.607. Correlation analysis of 13 morphological traits (4 qualitative, 9 quantitative) revealed significant positive correlations among three leaf-related traits and two inflorescence-related traits. Association analysis involving 40 SSR markers and the 13 morphological traits identified 39 significant marker–trait associations, comprising 18 SSR markers associated with 11 morphological traits. Among these SSR markers, 12 were associated with two to five quantitative or qualitative traits. Additionally, 10 SSR markers were significantly associated with three qualitative traits, while 15 SSR markers were associated with eight quantitative traits. Notably, GBPFM179, KNUPF59, and KNUPF167 were significantly associated with multiple quantitative or qualitative traits. GBPFM179 and KNUPF182 exhibited the highest R^2^ values, of 0.38, for stem color and days to maturity, respectively. These SSR markers demonstrate the potential for use in marker-assisted selection in *Perilla* breeding programs aimed at enhancing leaf or seed productivity through the selection of both quantitative and qualitative traits.

## 1. Introduction

*Perilla frutescens* (L.) Britt., a predominantly self-pollinating species within the Lamiaceae family (formerly Labiatae), is extensively cultivated and widely distributed across East Asia, with a significant prevalence in South Korea. The *Perilla* species includes two primary cultivated varieties, distinguished by morphology and usage: *P. frutescens* var. *frutescens* and *P. frutescens* var. *crispa*. In East Asian regions, *P. frutescens* var. *frutescens* is commonly grown for its oil-rich seeds and edible leaves and is referred to as “deulkkae” in Korean, “egoma” in Japanese, and “ren” in Chinese. Conversely, *P. frutescens* var. *crispa* is primarily used as a leafy vegetable and medicinal herb, known locally as “jaso” in Korean, “shiso” in Japanese, and “zisu” in Chinese [1,2]. These two cultivated *Perilla* varieties have been cultivated for centuries and are economically and culturally significant in East Asia, serving as sources of oil, vegetables, and medicinal products [2,3]. Despite clear morphological differences, *P. frutescens* var. *frutescens* and var. *crispa* are capable of hybridization through artificial pollination [4,5,6]. Both varieties possess the same chromosome number (2n = 40) [5,7], supporting their taxonomic classification as varieties of a single species. Weedy forms of the *Perilla* crop have been identified within both cultivated types of *P. frutescens*, as reported by Nitta and Ohnishi (1999) [8], Lee and Ohnishi (2001) [1], and Nitta et al. (2003) [2]. In *Perilla*, the cultivated var. *frutescens* is characterized by large, soft seeds (>2 mm), little to no seed dormancy, non-wrinkly green leaves, and a distinctive aroma associated with the Perilla ketone. In contrast, the weedy types, var. *crispa* and var. *frutescens*, possess small, hard seeds (<2 mm), strong seed dormancy, and either purple (var. *crispa*) or green (var. *frutescens*) leaves, which may be wrinkly or non-wrinkly. Their volatile profiles also differ, being predominantly associated with the Perilla aldehyde in var. *crispa* and with the Perilla ketone in weedy var. *frutescens* [1,2,3]. However, the cultivated var. *crispa*, which is mainly used in Japan, is morphologically indistinguishable from the weedy var. *crispa* found in South Korea [1]. Consequently, current analyses have yet to provide definitive evidence regarding the origins of these weedy forms. As such, further investigation is necessary to clearly differentiate between the two weedy types of *Perilla*.

Unraveling the molecular genetic basis of phenotypic variation remains a significant challenge, particularly because many agronomic traits, including yield, quality, and tolerance to biotic stress, are governed by quantitative trait loci (QTLs). Although QTLs typically underlie quantitative traits, they may also influence qualitative traits [9]. Identifying functional loci associated with these traits is critical for advancing marker-assisted selection (MAS) in crop-breeding programs. Genetic-mapping strategies, particularly linkage and association mapping analysis, are widely employed to analyze the genetic architectures of complex traits by correlating phenotypic variation with genetic polymorphisms. Among these, association mapping offers several advantages over traditional linkage analysis, including higher mapping resolution, broader allelic diversity, and reduced time to discovery [10,11,12,13]. However, the accuracy of association mapping depends on a comprehensive understanding of genetic diversity, population structure, and kinship within the study population. Generally, two primary approaches are used to identify genomic regions linked to important agronomic traits: QTL mapping, which is based on segregating populations derived from biparental crosses, and association mapping, which relies on linkage disequilibrium (LD) between markers and target traits [14,15,16]. Various types of mapping population have been developed in major crops for QTL studies, including backcross (BC) populations, F_2_ populations, doubled haploid (DH) populations, recombinant inbred line (RIL) populations, and near-isogenic line (NIL) populations [17,18,19,20]. However, in the case of *Perilla*, conducting QTL analysis using a genetic map from a separate population is very challenging because, unlike in other major crops (such as rice, wheat, and maize), various molecular markers, such as chromosome-specific simple sequence repeat (SSR) markers, have not yet been developed for the *Perilla* genome.

In genetic research, molecular marker-based techniques have been widely applied across various crop species to identify genomic regions associated with important agricultural traits. Among various DNA marker systems, SSRs are valued for their codominant nature; high polymorphism; reproducibility; and utility in genetic diversity and relationship analyses, population structure assessment, QTL detection, and association mapping [3,21,22,23]. Accordingly, SSRs are frequently employed in major crops such as rice [24], maize [18], and wheat [25] for population structure and association mapping studies. Although comprehensive SSR primer sets for *Perilla* are still lacking, recent studies have successfully utilized existing SSR markers to perform association mapping in accessions of both cultivated and weedy types of *Perilla* [3,6,21].

Therefore, this study aimed to develop SSR markers associated with both quantitative and qualitative traits in an F_2_ population derived from a cross between *P. frutescens* var. *crispa* and *P. frutescens* var. *frutescens*, using SSR primer sets developed specifically for *Perilla* species. The findings are expected to provide valuable information for future breeding programs targeting improved leafy-vegetable or seed varieties of *Perilla*.

## 2. Results

### 2.1. SSR Identification and Polymorphisms

In our study, we surveyed 200 SSR primer sets between the two parental lines of the F_2_ population of the *Perilla* crop. Based on the results, we selected 40 SSR primer sets that exhibited good amplification patterns and polymorphisms between the two parental lines of the F_2_ population (Supplementary Table S1). Then the 40 SSR primer sets were used to measure polymorphisms in terms of genetic diversity (GD), polymorphism information content (PIC), major allele frequency (MAF), and separation patterns of allele bands (SPABs) among the 68 individuals of the F_2_ population of the *Perilla* crop (Table 1). In the results, the GD ranged from 0.464 (KNUPF167) to 0.676 (KNUPF4), with an average of 0.607. The average PIC value was 0.537, ranging from 0.418 (KNUPF167) to 0.618 (KNUPF4). The MAF per locus varied from 0.400 (KNUPF36) to 0.700 (KNUPF167), with an average of 0.511.

In the case of the SPABs, five SSR primer sets (KWPE19, KNUPF36, KNUPF42, KNUPF61, KNUPF163) exhibited bias toward the AA genotype (parent A), while 15 SSR primer sets (KNUPF2, KNUPF3, KNUPF4, KNUPF15, KNUPF31, KNUPF40, KNUPF59, KNUPF93, KNUPF127, KNUPF156, KNUPF162, KNUPF168, KNUPF170, KNUPF182, KNUPF191) were skewed toward the BB genotype (parent B). Additionally, 14 SSR primer sets (GBPFM179, KNUPF12, KNUPF14, KNUPF16, KNUPF23, KNUPF29, KNUPF37, KNUPF39, KNUPF82, KNUPF83, KNUPF112, KNUPF130, KNUPF167, KNUPF176) showed bias toward the AB genotype (F_1_ hybrid). These deviations indicate that the corresponding SSR primer sets did not follow the expected Mendelian segregation ratio of 1:2:1 (AA:AB:BB) in the F_2_ population, as more than 50% of the allele bands were biased toward one of the two parents or the hybrid. The remaining six SSR primer sets (KWPE58, KNUPF9, KNUPF30, KNUPF50, KNUPF81, KNUPF169) generally followed the expected 1:2:1 Mendelian segregation ratio among the 68 analyzed F_2_ individuals, although this may not fully represent the segregation pattern of the entire F_2_ population. Meanwhile, among the SSR primer sets used in this analysis, six SSR primer sets (KNUPF2, KNUPF4, KNUPF82, KNUPF93, KNUPF163, KNUPF169) showed null band patterns in the 68 individuals of the F_2_ population (Table 1).

### 2.2. Phenotypic Variation and Association Analysis of 13 Qualitative and Quantitative Traits

**Figure 1:**
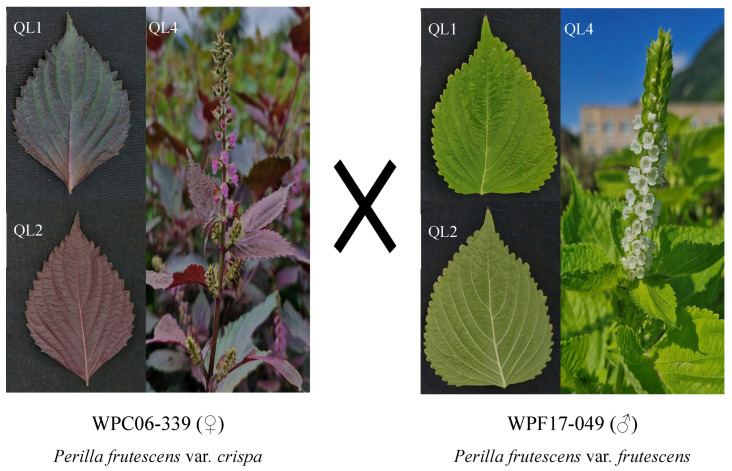
Morphological characteristics of the parental lines used for crossing: *Perilla frutescens* var. *crispa* (WPC06-339, ♀) and var. *frutescens* (WPF17-049, ♂). Figure previously published by Heo et al. (2025) [26].

The morphological characteristics of the 68 individuals of the F_2_ population were analyzed based on quantitative and qualitative traits that exhibited distinct differences between the two parental lines of the *Perilla* crop (Figure 1, Supplementary Table S2). As shown in Table 2, the distribution of the color of the leaf surfaces (QL1) in the F_2_ population was as follows: 67 individuals had green leaves, one individual had green–purple leaves, and none exhibited entirely purple leaves. For the color of leaf, reverse side (QL2), 18 individuals had green leaves, 36 had green–purple leaves, and 14 had purple leaves. The color of the stem (QL3) was green in six individuals, green–purple in 60 individuals, and purple in two individuals. The color of the flower (QL4) was white in 21 individuals, pink in 30 individuals, and purple in 17 individuals. For the quantitative traits for the 68 individuals of the F_2_ population, the days to heading (QN1) ranged from approximately 116 to 131 days, the days to flowering (QN2) from 125 to 141 days, and the days to maturity (QN3) from 152 to 173 days. Plant height (QN4) varied from 106.4 cm to 174.3 cm, length of inflorescence (QN5) ranged from 5.7 cm to 19.3 cm, and the number of florets (QN6) ranged from 28 to 64. Additionally, leaf length (QN7) varied from 9.6 cm to 15.0 cm, leaf width (QN8) from 6.2 cm to 10.9 cm, and leaf area (QN9) from 37.7 cm^2^ to 98.9 cm^2^.

**Figure 2:**
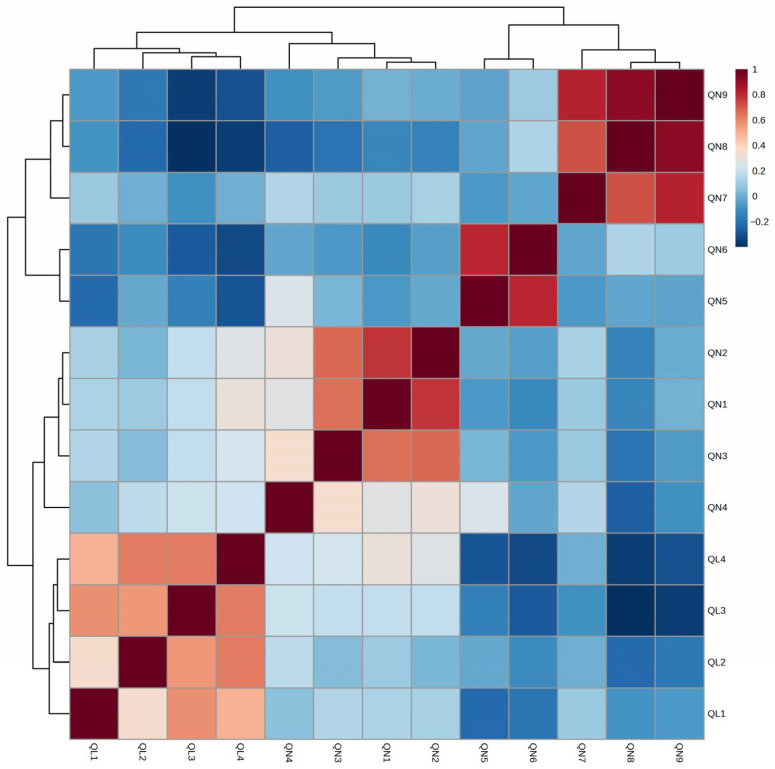
Correlation analysis among 13 morphological traits observed in the F_2_ population derived from a cross between *Perilla frutescens* var. *crispa* and var. *frutescens*.

Also, a correlation analysis was conducted on the 13 morphological traits observed in the 68 individuals of the F_2_ population (Figure 2, Table 2, Supplementary Table S3). That analysis identified strong positive correlations (r ≥ 0.7) among three leaf-related quantitative traits: leaf length (QN7), leaf width (QN8), and leaf area (QN9). Similarly, strong positive correlations were observed between inflorescence-related traits—length of inflorescence (QN5) and number of florets (QN6) (r = 0.811). Flowering time-related traits, including days to heading (QN1), days to flowering (QN2), and days to maturity (QN3), exhibited moderate-to-high positive correlations (r ≥ 0.6). For qualitative traits, positive correlations were found between color of stem (QL3) and both color of leaf surface (QL1) (r = 0.584) and color of leaf, reverse side (QL2) (r = 0.560). Additionally, flower color (QL4) was positively correlated with QL2 (r = 0.625) and QL3 (r = 0.627). Meanwhile, several negative correlations were also identified. Color of stem (QL3) was negatively correlated with number of florets (QN6) (r = −0.268), leaf width (QN8) (r = −0.396), and leaf area (QN9) (r = −0.353). Similarly, color of flower (QL4) showed negative correlations with length of inflorescence (QN5) (r = −0.290), QN6 (r = −0.314), QN8 (r = −0.352), and QN9 (r = −0.296) (Supplementary Table S3).

We surveyed 200 SSR markers and selected 40 that were polymorphic between the two parents (Supplementary Table S1, Supplementary Figure S1). These 40 SSR markers, along with phenotypic data for 13 qualitative and quantitative traits, were used to identify significant marker–trait associations (SMTAs) using TASSEL software. A total of 39 SMTAs involving 18 SSR markers associated with 11 morphological traits were detected using the GLM at a significance level of *p* ≤ 0.05 (Table 3). For leaf-related qualitative traits, eight SSR markers (GBPFM179, KNUPF4, KNUPF14, KNUPF23, KNUPF31, KNUPF59, KNUPF156, KNUPF167) were significantly associated with the color of the leaf surface (QL1). Color of leaf, reverse side (QL2) was significantly associated with KNUPF23 and KNUPF30, while the color of the stem (QL3) was significantly associated with GBPFM179, KNUPF59, and KNUPF112. Among the quantitative traits, the days to heading (QN1) were significantly associated with KNUPF16, KNUPF30, KNUPF40, and KNUPF59. The days to flowering (QN2) showed associations with KNUPF30, KNUPF59, and KNUPF182, and the days to maturity (QN3) were associated with KNUPF14, KNUPF16, KNUPF23, and KNUPF182. For reproductive traits, length of inflorescence (QN5) was associated with GBPFM179, KNUPF83, and KNUPF167, while the number of florets (QN6) was associated with GBPFM179, KNUPF37, KNUPF83, and KNUPF167. In terms of leaf-related quantitative traits, leaf length (QN7) was associated with KNUPF170. Leaf width (QN8) showed associations with KNUPF31, KNUPF93, KNUPF162, and KNUPF167. Leaf area (QN9) was associated with KNUPF93, KNUPF162, and KNUPF167. The coefficient of determination (R^2^) values of the significant associations ranged from 0.21 to 0.38, indicating that these markers explained a moderate proportion of the phenotypic variation in the F_2_ population. Notably, GBPFM179 and KNUPF182 showed the highest R^2^ values, of 0.38, for color of stem (QL3) and days to maturity (QN3), respectively (Table 3).

### 2.3. Genetic Verification of SSR Markers Among the F_2_ Population of Perilla

**Figure 3:**
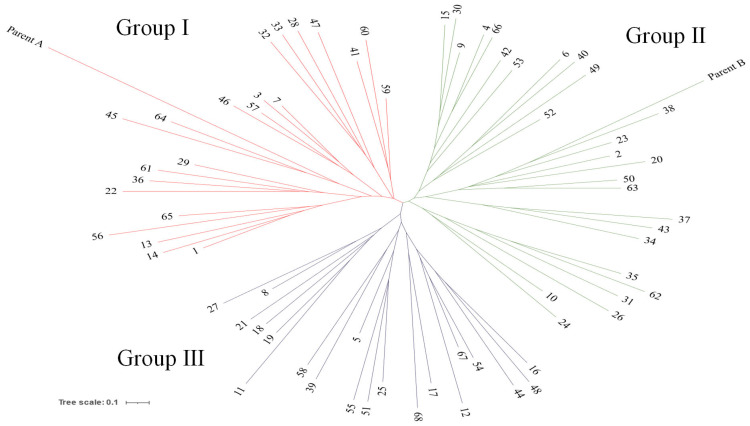
Neighbor-joining (NJ) tree constructed using 18 SSR markers for 68 F_2_ individuals and parental lines (parent A: *Perilla frutescens* var. *crispa*, parent B: *Perilla frutescens* var. *frutescens*).

To analyze the genetic relationships among 68 individuals from the F_2_ population, we constructed a neighbor-joining (NJ) tree using a total of 40 SSR markers (Supplementary Figure S2). The individuals were clustered into four major groups based on a genetic similarity of 51.4%. Group I consisted of 22 individuals, including the parental A line, while Group II comprised 21 individuals, including the parental B line. Group III contained 25 individuals, and Group IV included 2 individuals. Among the 68 individuals of the F_2_ population analyzed using 40 SSR markers, most of those clustered with the parental A and B lines in Groups I and II did not consistently exhibit the morphological characteristics of their respective parental lines, except in a few cases. Therefore, we conducted a genetic relationship analysis of these 68 F_2_ individuals using 18 SSR markers associated with 11 morphological traits, as identified through an association analysis based on the original 40 SSR markers. From the results, the 68 individuals of the F_2_ population were clustered into three major groups, with a genetic similarity of 53.4% (Figure 3). Group I consisted of 23 individuals, including the parental A line; Group II comprised 27 individuals, including the parental B line; and Group III contained 20 individuals. In the genetic relationship analysis using SSR markers associated with morphological characteristics, some individuals in the F_2_ population exhibited morphological traits similar to the parental lines of the crossbreeding combination. For example, within Group I, individuals 7, 22, 33, 36, and 56 showed similar morphological characteristics to parental line A in terms of leaf and stem color or flower color. Conversely, in Group II, individuals 2, 4, 9, 10, 15, 38, 42, 49, 52, and 53 displayed similar morphological traits to parental line B, including leaf and stem color or flower color. However, the remaining individuals in both Groups I and II did not consistently exhibit the same morphological characteristics as parental lines A and B. Additionally, most individuals in Group III showed intermediate morphological traits between parental lines A and B, although some exhibited features closely resembling one of the two parental lines.

## 3. Discussion

For genetic analysis in F_2_ populations, codominant markers are essential because they allow clear differentiation between homozygous and heterozygous genotypes. In *Perilla frutescens*, research on the development and application of SSR markers is currently in progress [21,23,27]. SSR markers, in particular, offer several advantages over other marker systems. First, they provide high reproducibility, which is crucial for reliable genetic studies. Second, the hypervariable nature of SSRs generates substantial allelic diversity, even among closely related varieties, resulting in abundant polymorphic genetic information. Third, the codominant inheritance of SSR polymorphisms enables the detection of both homozygous and heterozygous alleles, making them highly suitable for genetic analyses, such as segregation studies in F_2_ populations; DH, NILs, or RILs; and pedigree analysis in hybrids [20,21,22]. Consequently, the application of SSR markers in analyzing genetic diversity, determining genetic relationships, and conducting association mapping can facilitate the identification of novel molecular markers linked to target traits, thereby advancing the development of desirable varieties and lines in *Perilla* crop-breeding programs.

In this study, an F_2_ population consisting of 68 individuals was generated by crossing WPC06-339 (female parent, weedy var. *crispa*) and WPF17-049 (male parent, weedy var. *frutescens*) with the goal of identifying molecular markers associated with morphological traits that differ between the two parental lines (Figure 1, Supplementary Table S2). The F_2_ individuals exhibited continuous variation across both qualitative and quantitative morphological traits, with certain traits falling within the parental phenotypic ranges, while others displayed transgressive segregation beyond those ranges (Table 2). Specifically, traits such as color of leaf, reverse side (QL2) and color of flower (QL4) segregated in a 1:2:1 Mendelian ratio, indicating monogenic inheritance likely governed by single major genes [28]. Conversely, traits including color of leaf surface (QL1) and color of stem (QL3) exhibited non-Mendelian separation patterns, suggesting the involvement of multiple genes and more complex inheritance mechanisms [29]. Additionally, nine quantitative traits showed broad phenotypic distributions, including cases of transgressive segregation, consistent with polygenic control involving additive and/or epistatic gene interactions [30]. These findings suggest that morphological trait inheritance in *Perilla* is highly trait-specific and governed by diverse genetic mechanisms. Similar patterns have been reported in a previous study by Lim et al. (2021) [6], who observed variable segregation behaviors across morphological traits in a *Perilla* F_2_ population. These observations are also consistent with the findings of Scheid (2022) [31], who emphasized that while certain traits in an F_2_ population exhibited Mendelian inheritance, others deviated due to polygenic control, gene interactions, and environmental influences. Collectively, these results underscore the necessity of considering both monogenic and polygenic mechanisms when investigating morphological trait inheritance in *Perilla* and related crop species.

We also performed a correlation analysis for the 13 morphological traits observed in the 68 individuals of the F_2_ population (Figure 2, Table 2, Supplementary Table S3). The results showed strong positive correlations (r ≥ 0.7) among the three leaf-related quantitative traits (QN7, QN8, QN9). This pattern is consistent with previous findings in *Perilla frutescens*, where traits such as leaf length, leaf width, and leaf area exhibited similar interrelationships [6,21]. In contrast, negative correlations were observed between certain qualitative plant traits (e.g., QL3 and QL4) and quantitative traits related to inflorescence and leaf size. Specifically, QL3 showed negative correlations with QN6 (r = −0.268), QN8 (r = −0.396), and QN9 (r = −0.353), with similar patterns observed for QL4. These negative associations may reflect developmental trade-offs between vegetative growth and reproductive development, as reported in other plant species such as *Arabidopsis thaliana* and maize [12,32]. Collectively, these findings suggest potential genetic linkages or pleiotropic effects influencing the coordinated regulation of traits related to leaf morphology and reproductive structures.

Furthermore, to gain a clearer understanding of the genetic relationships among the 68 individuals of the F_2_ population, we conducted a genetic analysis of them and their two parental lines using 40 SSR markers. The NJ tree analysis clustered the 68 F_2_ individuals and the two parental lines into four major groups. Most individuals in Groups I and II, which clustered with the parental A line and parental B line, respectively, did not consistently exhibit the morphological characteristics of their respective parental lines, with some exceptions (Supplementary Figure S2). This inconsistency is consistent with previous reports that SSR-based genetic clustering often fails to fully reflect phenotypic variation, as SSR markers capture genome-wide polymorphisms rather than loci directly controlling morphological traits, which are typically governed by polygenic inheritance and environmental effects [22,33]. Therefore, to enhance the resolution of genotype–phenotype relationships, we conducted a targeted genetic analysis using 18 SSR markers significantly associated with 11 morphological traits, identified through association analysis based on the original 40 SSR markers, following methodologies commonly used in marker–trait association studies [3,21,33]. In these results, the 68 F_2_ individuals were clustered into three major groups with a genetic similarity of 53.4% (Figure 3). Some F_2_ individuals exhibited morphological traits resembling those of the parental lines of the F_2_ population. For instance, in Group I, individuals 7, 22, 33, 36, and 56 displayed morphological characteristics similar to parental line A, particularly in leaf and stem color and flower color. In contrast, Group II included individuals 2, 4, 9, 10, 15, 38, 42, 49, 52, and 53, which showed traits resembling parental line B, including leaf and stem color and flower color. Group III represented a mix of individuals with genetic characteristics from both parental lines, indicating potential recombination events. Previous studies in crops such as rice, maize, and soybeans have emphasized that phenotypically diverse F_2_ populations serve as valuable genetic resources for identifying trait-linked markers through linkage mapping and QTL analysis [18,34,35,36]. However, although F_2_ populations are useful for QTL mapping and initial marker discovery, genetic mapping and QTL analysis become difficult in species where the chromosomal locations of markers are unknown, such as in *Perilla* crops [27]. Therefore, the identified groups of F_2_ individuals, along with their leaf, stem, and flower color-related traits, are considered valuable genetic resources for identifying molecular markers associated with these morphological characteristics in *Perilla* crops through association mapping analysis.

Meanwhile, association mapping has been suggested as an effective approach for identifying loci associated with complex traits [36,37]. In this study, we developed an F_2_ population for performing association mapping analysis to find molecular markers associated with both quantitative and qualitative traits of *Perilla* crops. We employed SSR markers for the analysis because of their high polymorphism and codominant nature [22,23,27,38]. Notably, SSR markers are particularly effective in detecting the segregation patterns of allelic bands in an F_2_ population. In our study, 40 SSR primer sets exhibited clear amplification patterns and polymorphisms among the 68 individuals in the F_2_ population (Table 1, Supplementary Figure S1). Among these, 5 SSR primer sets displayed bias toward the AA genotype (parent A), 15 SSR primer sets were skewed toward the BB genotype (parent B), and 14 SSR primer sets showed bias toward the AB genotype (F_1_ hybrid). Although this may not fully represent the segregation pattern of the entire F_2_ population, the observed segregation ratios for these SSR primer sets deviate from the expected Mendelian ratio of 1:2:1 (AA:AB:BB). Despite this deviation, the SSR markers were effective in revealing the genotypic segregation patterns of AA, BB, and AB alleles in the F_2_ population, thereby providing valuable insights for the genetic analysis of both homozygous and heterozygous individuals. In addition, six SSR primer sets exhibited one to six null bands in the F_2_ population. These null bands, which have also been reported previously (Yazdani et al., 2003) [39], are likely due to mutations in the microsatellite primer-binding regions that inhibit amplification and result in missing PCR products.

To identify SSR markers associated with both quantitative and qualitative traits in the F_2_ population, we conducted a marker–trait association (SMTA) analysis using TASSEL software. This analysis involved examining the relationships between 40 SSR markers and 13 morphological traits (4 qualitative and 9 quantitative) in 68 F_2_ individuals. Based on these results, we identified 39 SMTAs involving 18 SSR markers associated with 11 morphological traits, as determined using the GLM at a significance level of *p* ≤ 0.05 (Table 3). These results revealed significant associations between SSR markers and key morphological traits in *Perilla*. For qualitative traits, 10 SSR markers (GBPFM179, KNUPF4, KNUPF14, KNUPF23, KNUPF30, KNUPF31, KNUPF59, KNUPF112, KNUPF156, KNUPF167) were linked to color of leaf surface (QL1); color of leaf, reverse side (QL2); and color of stem (QL3). Regarding quantitative traits, 15 SSR markers (GBPFM179, KNUPF14, KNUPF16, KNUPF23, KNUPF30, KNUPF31, KNUPF37, KNUPF40, KNUPF59, KNUPF83, KNUPF93, KNUPF162, KNUPF167, KNUPF170, KNUPF182) were linked to days to heading (QN1), days to flowering (QN2), days to maturity (QN3), length of inflorescence (QN5), number of florets (QN6), leaf length (QN7), leaf width (QN8), and leaf area (QN9). These results suggest that the identified SSR markers are valuable resources for developing molecular markers associated with color traits and key agronomic characteristics in *Perilla* crops. Our findings indicate that most quantitative and qualitative traits, except for QL4 and QN4, were associated with one to five SSR markers, depending on the specific characteristics. Among these, 12 SSR markers (GBPFM179, KNUPF14, KNUPF16, KNUPF23, KNUPF30, KNUPF31, KNUPF59, KNUPF83, KNUPF93, KNUPF162, KNUPF167, KNUPF182) were significantly associated with two to five traits, including three qualitative traits (QL1, QL2, QL3) and seven quantitative traits (QN1, QN2, QN3, QN5, QN8, QN9) (Table 3). Notably, GBPFM179 (QL1, QL3, QN5, QN6) and KNUPF59 (QL1, QL3, QN1, QN2) were each significantly associated with four traits, while KNUPF167 exhibited associations with five traits (QL1, QN5, QN6, QN8, QN9). These findings are consistent with previous reports for crops such as rice, maize, and soybeans, where SSR markers associated with multiple morphological and agronomic traits have been identified. For example, Zhang et al. (2012) [16], Wang et al. (2016) [40], and Galal et al. (2025) [41] reported SSR markers simultaneously linked to traits like plant height, panicle length, tiller number, and grain yield in rice and maize. Similarly, in soybeans, Li et al. (2023) [42] identified SSR markers associated with both plant height and the growth period, supporting their utility for multi-trait selection in marker-assisted selection (MAS). In comparison, the present study in *Perilla* identified 12 SSR markers associated with two to five traits each, spanning both qualitative (e.g., stem and leaf color) and quantitative traits (e.g., flowering time, leaf area, days to maturity). Markers such as GBPFM179, KNUPF59, and KNUPF167 demonstrated multi-trait associations comparable with those observed in major crop species [40,41,42]. The R^2^ values for these associations ranged from 0.21 to 0.38, indicating that the identified SSR markers explained a moderate proportion of phenotypic variation. This is consistent with previous studies of rice and maize, where SSR-trait associations typically yielded R^2^ values between 0.10 and 0.40, depending on population structure and trait complexity [36]. In particular, GBPFM179 and KNUPF182 exhibited the highest R^2^ values (0.38), suggesting relatively strong marker–trait associations (Table 3). Additionally, several SSR markers previously reported in the literature also exhibited significant associations in the present study. Specifically, KNUPF4, KNUPF16, and KNUPF31 were associated with seed-related traits [3,6], whereas KNUPF23, KNUPF30, and KNUPF37 were associated with leaf- and stem-related traits [3]. While prior studies predominantly reported markers associated with qualitative traits such as seed and leaf characteristics, the current study identified markers associated with both qualitative and quantitative traits, including flowering time, leaf width, leaf area, and other plant-related traits. These findings suggest that these SSR markers will be valuable for selecting quantitative and qualitative traits in *Perilla* crop-breeding programs.

In this study, we identified SSR markers associated with both quantitative and qualitative traits using an F_2_ population derived from a cross between WPC06-339 (female parent, weedy var. *crispa*) and WPF17-049 (male parent, weedy var. *frutescens*). Notably, the molecular markers linked to quantitative traits related to plant and leaf characteristics are expected to provide valuable insights for molecular breeding aimed at improving leaf quality and productivity in *Perilla* crops in South Korea. Further genome-wide analyses at the chromosomal level in *Perilla* crops could provide a more comprehensive understanding of SSR markers associated with specific quantitative and qualitative traits in *Perilla*. Previous taxonomic studies, as discussed in the Introduction, have attempted to differentiate the two weedy types of *Perilla* based on morphological traits and DNA markers. However, distinguishing these *Perilla* types remains challenging because of the presence of intermediate forms, such as weedy hybrids arising from inter-varietal crosses or escape forms derived from cultivated *Perilla* varieties. The SSR markers identified in this study, associated with both quantitative and qualitative traits, have the potential to be effective tools for distinguishing between the two weedy types of *Perilla* crop. Additionally, these SSR markers may prove useful for identifying genetic diversity, constructing genetic linkage maps, and identifying key genes or QTLs for breeding programs aimed at enhancing leaf quality, plant vigor, and seed or leaf yield through marker-assisted selection in *Perilla* crops.

## 4. Materials and Methods

### 4.1. Plant Materials and Morphological Characteristics of F_2_ Population

A total of 68 individuals from the F_2_ population of the *Perilla* crop from a cross between WPC06-339 (female parent) and WPF17-049 (male parent) were selected (Table 2). The female parent, WPC06-339, is a weedy type of var. *crispa* characterized by non-wrinkled leaves with a green–purple surface and a purple reverse side, a purple stem, and a fragrance specific to var. *crispa* (Figure 1, Supplementary Table S2). The male parent, WPF17-049, is a weedy type of var. *frutescens* with non-wrinkled leaves that have a green surface and reverse side, a green stem, and a fragrance specific to var. *frutescens* (Figure 1, Supplementary Table S2). Morphological data for the 68 F_2_ individuals were obtained from a previous study by Heo et al. (2025) [26]. These individuals, selected to develop SSR molecular markers, represent the morphological characteristics distinguishing the parental lines. The morphological characteristics of the F_2_ population were evaluated based on both qualitative and quantitative traits associated with the parental lines (Figure 1, Table 2). The plant morphologies of the parent lines are illustrated in Figure 1. Four qualitative traits, namely color of leaf surface (QL1); color of leaf, reverse side (QL2); color of stem (QL3); and color of flower (QL4), and nine quantitative traits, namely days to heading (QN1), days to flowering (QN2), days to maturity (QN3), plant height (QN4), length of inflorescence (QN5), number of florets (QN6), leaf length (QN7), leaf width (QN8), and leaf area (QN9), that were investigated in the 68 individuals of the F_2_ population in the previous study by Heo et al. (2025) [26] are shown in Table 2.

### 4.2. DNA Extraction and SSR Analysis

Genomic DNA was isolated from young leaf tissue using the CTAB method. DNA quality and concentration were assessed using a spectrophotometer. We surveyed a total of 200 SSR *Perilla* primer sets [27] and selected 40 SSR loci representing the polymorphism between the two parents (Supplementary Table S1). Each SSR marker was amplified in a 20 µL polymerase chain reaction (PCR) containing 20 ng of genomic DNA, 1× PCR buffer, 0.5 µM of each primer (forward and reverse), 0.2 mM dNTPs, and 1 unit of Taq DNA polymerase (Biotools, Madrid, Spain). The PCR cycling conditions included an initial denaturation at 94 °C for 5 min, followed by 35 cycles consisting of denaturation at 94 °C for 30 s, annealing at 55 °C for 30 s, and extension at 72 °C for 1 min and 30 s, with a final extension at 72 °C for 5 min. Following PCR amplification, amplified products were separated by electrophoresis using a miniature vertical electrophoresis system (MGV-202–33; CBS Scientific Company, San Diego, CA, USA). Each PCR product (3 µL) was mixed with 3 µL of a loading buffer containing 98% formamide, 0.02% xylene cyanol, 0.02% bromophenol blue, and 5 mM NaOH. After denaturation and rapid cooling, 2 µL of the mixture was loaded onto a 6% denaturing polyacrylamide gel (7.5 M urea; 19:1 acrylamide:bisacrylamide) and run in 0.5× TBE buffer at 250 V for 30 min. DNA fragments were visualized using ethidium bromide staining.

### 4.3. Data Analysis

Morphological data of the F_2_ population were analyzed using MetaboAnalyst 6.0 to generate a correlation matrix among traits based on the Pearson correlation method. For qualitative traits, ordinal grade values were assigned to each category (e.g., QL1–QL3: green = 1, green–purple = 2, purple = 3; QL4: white = 1, pink = 2, purple = 3) to enable numerical analysis. Quantitative traits were analyzed using their directly measured values. DNA fragments amplified by the 40 *Perilla* SSR primer sets in the 68 individuals of the F_2_ population were scored as either present (1) or absent (0). Genetic diversity (GD) for each group of accessions was calculated using Nei’s formula [43]:

GD = 1 ∑ *P_i_*^2^,

where *Pi* is the frequency of the *i*th SSR allele within a group. For the 68 individuals of the F_2_ population and the 40 SSR loci, the numbers of alleles, major allele frequencies (MAF), and polymorphism information content (PIC) were calculated using PowerMarker version 3.25 [44]. Genetic similarities between all pairs of the F_2_ individuals of the *Perilla* crop were estimated based on Euclidean distances derived from SSR genotype data. To analyze the clustering patterns among individuals of the F_2_ population, a neighbor-joining (NJ) tree was constructed using the ape package in R version 4.3.2 [45]. Bootstrapping with 10,000 replicates was performed in R, and the resulting tree was visualized using the Interactive Tree Of Life (iTOL) online platform [46]. Association analysis was performed using morphological data from the 68 F_2_ individuals, encompassing four qualitative and nine quantitative traits (Table 2). Marker–trait associations were examined using the general linear model (GLM) implemented in TASSEL 3.0 [47]. A permutation test with 10,000 iterations was employed to evaluate marker significance at a threshold of *p* ≤ 0.05.

## 5. Conclusions

This study aimed to identify molecular markers associated with quantitative and qualitative traits in *Perilla frutescens* by analyzing an F_2_ population derived from a cross between two weedy types: *P. frutescens* var. *crispa* and *P. frutescens* var. *frutescens*. The objective was to identify markers linked to key morphological traits that could aid in distinguishing weedy and cultivated forms and facilitate marker-assisted breeding. A total of 13 morphological traits (4 qualitative and 9 quantitative) and 40 SSR primer sets were employed for genetic analysis. GD ranged from 0.464 to 0.676, with an average of 0.607. Among the SSR primer sets, 5 (KWPE19, KNUPF36, KNUPF42, KNUPF61, KNUPF163) were biased toward the AA genotype (parent A), while 15 SSR primer sets (including KNUPF2, KNUPF3, KNUPF4, KNUPF15, and KNUPF59) exhibited bias toward the BB genotype (parent B). Correlation analysis of the 13 morphological traits revealed strong positive correlations among three leaf-related traits (QN7, QN8, QN9) and two inflorescence-related traits (QN5, QN6). Association analysis revealed 39 SMTAs involving 18 SSR markers linked to 11 morphological traits (3 qualitative, 8 quantitative). The F_2_ population was divided into three major groups based on the SSR markers. Group I included individuals genetically aligned with the parent A line, while Group II consisted of those closely related to the parent B line. Group III represented a mix of individuals with genetic characteristics from both parental lines. Among the SSR markers, 12 SSR markers were associated with multiple traits; in particular, GBPFM179, KNUPF59, and KNUPF167 were significantly linked to four or five of both quantitative and qualitative characteristics. Additionally, 10 SSR markers were associated with three qualitative traits (QL1, QL2, QL3), while 15 markers were linked to eight quantitative traits (QN1, QN2, QN3, QN5, QN6, QN7, QN8, QN9). These SSR markers will be valuable for distinguishing cultivated and weedy *Perilla* types and for marker-assisted selection in breeding programs targeting leaf or seed productivity.

## Figures and Tables

**Table 1:** Estimates of GD, PIC, MAF, and the separation patterns of allele bands (SPABs) for 40 SSR markers used for 68 individuals of the F_2_ *Perilla* population.

Marker	GD	PIC	MAF	Separation of the F_2_ Population			
				AA	AB	BB	NULL
GBPFM179	0.523	0.468	0.643	12	45	11	0
KWPE19	0.625	0.552	0.486	21	34	13	0
KWPE58	0.625	0.555	0.500	17	35	16	0
KNUPF2	0.599	0.519	0.500	7	35	25	1
KNUPF3	0.608	0.531	0.500	10	35	23	0
KNUPF4	0.676	0.618	0.429	10	30	22	6
KNUPF9	0.630	0.559	0.486	15	34	19	0
KNUPF12	0.570	0.507	0.586	15	41	12	0
KNUPF14	0.569	0.504	0.586	11	41	16	0
KNUPF15	0.644	0.570	0.443	15	31	22	0
KNUPF16	0.589	0.522	0.557	17	39	12	0
KNUPF23	0.570	0.507	0.586	15	41	12	0
KNUPF29	0.597	0.528	0.543	18	38	12	0
KNUPF30	0.622	0.551	0.500	14	35	19	0
KNUPF31	0.606	0.525	0.486	9	34	25	0
KNUPF36	0.650	0.575	0.400	25	28	15	0
KNUPF37	0.601	0.534	0.543	15	38	15	0
KNUPF39	0.601	0.534	0.543	15	38	15	0
KNUPF40	0.620	0.548	0.500	13	35	20	0
KNUPF42	0.643	0.567	0.429	24	30	14	0
KNUPF50	0.616	0.546	0.514	14	36	18	0
KNUPF59	0.640	0.565	0.443	14	31	23	0
KNUPF61	0.630	0.555	0.471	22	33	13	0
KNUPF81	0.653	0.580	0.429	19	30	19	0
KNUPF82	0.563	0.505	0.600	15	42	10	1
KNUPF83	0.558	0.497	0.600	11	42	15	0
KNUPF93	0.639	0.569	0.471	13	33	21	1
KNUPF112	0.495	0.430	0.657	5	46	17	0
KNUPF127	0.626	0.550	0.471	12	33	23	0
KNUPF130	0.591	0.524	0.557	13	39	16	0
KNUPF156	0.592	0.516	0.529	9	37	22	0
KNUPF162	0.625	0.552	0.486	13	34	21	0
KNUPF163	0.661	0.591	0.414	22	29	16	1
KNUPF167	0.464	0.418	0.700	8	49	11	0
KNUPF168	0.640	0.565	0.443	14	31	23	0
KNUPF169	0.645	0.578	0.471	17	33	17	1
KNUPF170	0.608	0.531	0.500	10	35	23	0
KNUPF176	0.570	0.507	0.586	15	41	12	0
KNUPF182	0.630	0.555	0.471	13	33	22	0
KNUPF191	0.656	0.582	0.400	17	28	23	0
Max	0.676	0.618	0.700				
Min	0.464	0.418	0.400				
Mean	0.607	0.537	0.511				

GD: genetic diversity; PIC: polymorphism information content; MAF: major allele frequency; AA: homozygous for Parent A allele; AB: heterozygous; BB: homozygous for parent B allele; NULL: missing genotype data.

**Table 2:** Morphological characteristics of two parental lines and 68 F_2_ individuals derived from a cross between *Perilla frutescens* var. *crispa* (Parent A) and var. *frutescens* (Parent B).

	QL1	QL2	QL3	QL4	QN1	QN2	QN3	QN4	QN5	QN6	QN7	QN8	QN9
Parent A	G/P	Purple	Purple	Purple	124	134	168	151.4	8.6	33.3	12.6	8.3	59.8
Parent B	Green	Green	Green	White	119	126	157	105.4	8.5	44	12.6	11.2	88.8
1	Green	G/P	G/P	Pink	119	131	164	149.8	12.7	48	11.1	7.1	50.6
2	Green	Green	Green	White	116	130	160	139.4	12.2	52	11.6	8.6	62.4
3	Green	G/P	G/P	Pink	121	131	161	143.2	14.5	56	10.8	7.2	46.5
4	Green	Green	G/P	White	122	132	166	154.8	14.7	52	10.9	7.5	49.2
5	Green	Green	Green	White	122	136	165	174.3	13.8	52	12.4	9.1	71.5
6	Green	Green	G/P	Pink	121	133	171	148.7	9.1	32	11.6	8.9	64.9
7	Green	Purple	G/P	Purple	121	130	152	130.8	13.3	44	11.5	9.2	64.9
8	Green	Purple	Purple	Purple	119	129	158	159.8	6	32	12.2	8.3	58.6
9	Green	G/P	G/P	White	120	128	159	120.2	9.2	40	10.2	7.8	48.0
10	Green	G/P	G/P	White	116	125	157	150.6	13.8	44	11.8	8.9	67.7
11	Green	Green	G/P	Pink	123	133	164	163.9	10.5	36	11.9	8.1	56.9
12	Green	Green	G/P	Pink	124	128	166	158.7	11.7	44	13.2	9.2	75.7
13	Green	Green	G/P	Pink	121	132	160	165.4	15.4	48	11.6	9.2	64.3
14	Green	Green	G/P	Pink	125	133	161	133.4	11.5	48	11.5	8.7	64.3
15	Green	Green	G/P	White	116	126	158	149.4	8.6	32	11.4	9.0	63.2
16	Green	Purple	G/P	White	116	128	157	106.4	12.3	52	11.5	10.5	75.5
17	Green	Green	Green	White	123	131	159	171.4	13.3	44	11.5	8.9	64.6
18	Green	G/P	G/P	Pink	121	132	161	134.7	11.6	48	10.5	7.4	47.0
19	Green	G/P	G/P	White	117	129	159	156.3	13.4	48	10.8	8.1	55.8
20	Green	G/P	G/P	Pink	123	135	164	138.7	11.6	52	12.0	8.3	63.9
21	Green	G/P	G/P	Purple	121	131	164	149.2	12.8	44	10.0	6.2	39.5
22	Green	Purple	G/P	Purple	125	131	163	140.6	9.9	36	11.0	7.0	46.3
23	Green	G/P	G/P	Pink	121	131	166	160.8	11.8	40	11.8	7.7	53.0
24	Green	Purple	G/P	Purple	120	128	159	174.2	8.2	28	11.4	7.9	53.3
25	Green	Purple	G/P	Purple	122	131	167	174.1	10.5	40	13.0	8.3	65.3
26	Green	Green	G/P	Pink	125	135	166	164.1	10.6	40	12.1	7.8	56.3
27	Green	G/P	G/P	Pink	121	133	165	158.4	10.6	44	11.4	6.9	46.1
28	Green	G/P	G/P	Pink	119	130	160	144.4	7.4	28	11.4	7.0	37.7
29	Green	G/P	G/P	Pink	120	130	161	134.7	9.7	44	12.1	8.8	41.3
30	Green	G/P	G/P	Purple	118	128	162	141.1	14.3	48	12.4	9.3	46.1
31	Green	G/P	G/P	Pink	124	135	165	163.2	12.2	44	12.5	9.1	69.2
32	Green	G/P	G/P	White	117	129	161	134.8	18.6	60	11.7	7.7	53.7
33	Green	G/P	G/P	Purple	121	136	171	138.7	8.8	36	12.4	8.3	60.0
34	Green	Purple	G/P	Pink	121	131	166	161.9	13.5	44	12.0	7.9	55.4
35	Green	G/P	G/P	Pink	120	129	160	154.3	9.2	28	12.1	7.8	57.3
36	Green	Green	G/P	Purple	124	135	167	143.1	9.6	44	10.8	7.8	51.1
37	Green	G/P	G/P	Purple	124	132	160	154.9	11.4	44	11.5	7.3	49.6
38	Green	Green	Green	White	120	130	164	164.9	16.2	56	12.1	8.9	63.9
39	Green	Green	G/P	White	120	131	171	156.3	14	44	11.7	6.9	46.7
40	Green	G/P	G/P	Pink	121	131	163	165.8	15.6	52	11.9	8.9	63.2
41	Green	Green	Green	White	125	134	170	164.3	10.7	52	13.5	10.4	86.5
42	Green	G/P	G/P	White	123	133	162	172.7	17.9	52	12.0	9.3	69.3
43	Green	Purple	G/P	Purple	121	132	163	170.9	19.2	64	15.0	10.2	87.6
44	Green	G/P	G/P	Pink	125	135	163	166.2	13.8	44	12.7	8.9	69.4
45	Green	G/P	G/P	Pink	125	132	167	157.3	15	44	13.2	8.8	68.4
46	Green	G/P	Green	White	121	132	166	146.3	13.1	40	14.7	10.9	95.6
47	Green	G/P	G/P	Pink	127	137	161	145.8	5.7	28	14.6	9.9	88.1
48	Green	G/P	G/P	Pink	122	135	166	168.3	11.9	44	12.7	9.0	72.9
49	Green	G/P	G/P	White	121	130	161	159.6	9.8	28	13.0	9.9	80.8
50	Green	Purple	G/P	Purple	124	132	168	167.7	11.5	48	14.9	10.6	98.9
51	Green	G/P	G/P	Pink	124	133	169	154.3	13.8	56	13.6	10.2	86.5
52	Green	Green	G/P	White	121	131	167	150.3	8.5	28	13.5	10.2	86.2
53	Green	G/P	G/P	White	126	132	172	154.2	15.7	48	11.5	8.1	57.7
54	Green	Green	G/P	White	124	131	166	150.3	15.4	48	11.5	8.2	55.1
55	Green	Purple	G/P	Purple	126	134	173	170.3	10.2	36	11.0	7.5	49.6
56	Green	Purple	G/P	Purple	125	131	172	153.8	14.3	48	9.6	6.6	38.3
57	Green	Green	G/P	White	124	135	167	154.5	15	56	13.2	9.9	79.0
58	Green	G/P	G/P	White	120	131	161	139.6	19.3	64	12.7	9.6	75.6
59	Green	G/P	G/P	Pink	128	137	170	137.2	12.1	44	12.4	9.1	68.8
60	Green	G/P	G/P	Pink	121	133	168	149.1	11.6	48	12.1	10.2	76.0
61	Green	G/P	G/P	Pink	128	137	173	153.2	12.6	44	12.6	9.5	73.3
62	Green	G/P	G/P	Purple	121	131	166	154.3	7.9	28	14.0	8.8	75.9
63	Green	G/P	G/P	Purple	125	134	166	154.2	14	44	10.7	6.5	41.5
64	Green	G/P	G/P	Pink	126	135	172	140.1	13.3	40	11.1	7.8	53.2
65	Green	G/P	G/P	Pink	131	141	173	147.3	7.9	32	11.1	7.8	54.1
66	Green	Purple	G/P	Pink	125	131	166	164.1	12.8	48	11.1	7.6	53.5
67	G/P	Purple	Purple	Purple	123	131	164	165.8	10.9	48	12.1	7.9	56.8
68	Green	Purple	G/P	Pink	120	130	161	169.3	17.3	48	11.6	8.2	57.0
Max					131	141	173	174.3	19.3	64	15.0	10.9	98.9
Min					116	125	152	106.4	5.7	28	9.6	6.2	37.7
Mean					122	132	164	153.1	12.3	44.1	12.0	8.5	62.2

**Table 3:** Significant SSR markers associated with 13 morphological traits based on GLM analysis.

Trait	Marker	*p* Value	Marker R^2^	Trait	Marker	*p* Value	Marker R^2^
QL1	GBPFM179	0.01	0.31	QN3	KNUPF14	0.04	0.21
	KNUPF4	0.04	0.22		KNUPF16	0.02	0.26
	KNUPF14	0.04	0.22		KNUPF23	0.03	0.25
	KNUPF23	0.04	0.22		KNUPF182	0.00	0.38
	KNUPF31	0.04	0.22	QN5	GBPFM179	0.02	0.27
	KNUPF59	0.04	0.22		KNUPF83	0.04	0.22
	KNUPF156	0.04	0.22		KNUPF167	0.01	0.28
	KNUPF167	0.04	0.22	QN6	GBPFM179	0.04	0.21
QL2	KNUPF23	0.02	0.25		KNUPF37	0.04	0.21
	KNUPF30	0.04	0.22		KNUPF83	0.02	0.27
QL3	GBPFM179	0.00	0.38		KNUPF167	0.01	0.29
	KNUPF59	0.01	0.29	QN7	KNUPF170	0.04	0.21
	KNUPF112	0.02	0.26	QN8	KNUPF31	0.04	0.22
QN1	KNUPF16	0.01	0.28		KNUPF93	0.03	0.24
	KNUPF30	0.01	0.29		KNUPF162	0.03	0.24
	KNUPF40	0.03	0.23		KNUPF167	0.03	0.24
	KNUPF59	0.01	0.32	QN9	KNUPF93	0.04	0.22
QN2	KNUPF30	0.02	0.27		KNUPF162	0.04	0.22
	KNUPF59	0.02	0.25		KNUPF167	0.03	0.23
	KNUPF182	0.01	0.33				

## Data Availability

All data generated or analyzed during this study are included in this published article and its supplementary information files.

## Supplementary Materials

The following supporting information can be downloaded at: https://www.mdpi.com/article/10.3390/plants14172799/s1, Figure S1: Genotyping pattern of SSR marker KNUPF83 in 68 F_2_ individuals and the two parental lines of *Perilla frutescens.* Figure S2. Neighbor-joining (NJ) tree constructed using 40 SSR markers for 68 F_2_ individuals and parental lines (Parent A: *Perilla frutescens* var. *crispa*, Parent B: *Perilla frutescens* var. *frutescens*). Table S1. List of SSR markers used in this study with their sequence information and repeat motifs. Table S2. Morphological traits and their measurements in Parent A (var. *crispa*) and Parent B (var. *frutescens*). Table S3. Pearson correlation coefficients among 13 morphological traits observed in the F_2_ population of *Perilla frutescens*.
