# Supplementary material for: Association Mapping Analysis of Morphological Characteristics in F2 Population of Perilla (Perilla frutescens L.) Using SSR Markers
Source: Plants (Basel). 2025 Sep 6;14(17):2799. doi: 10.3390/plants14172799 (PMC12430519; doi:10.3390/plants14172799)
Supplement: Supplementary file 1 [file plants-14-02799-s001.zip › Supplementary Figure S1.pdf]

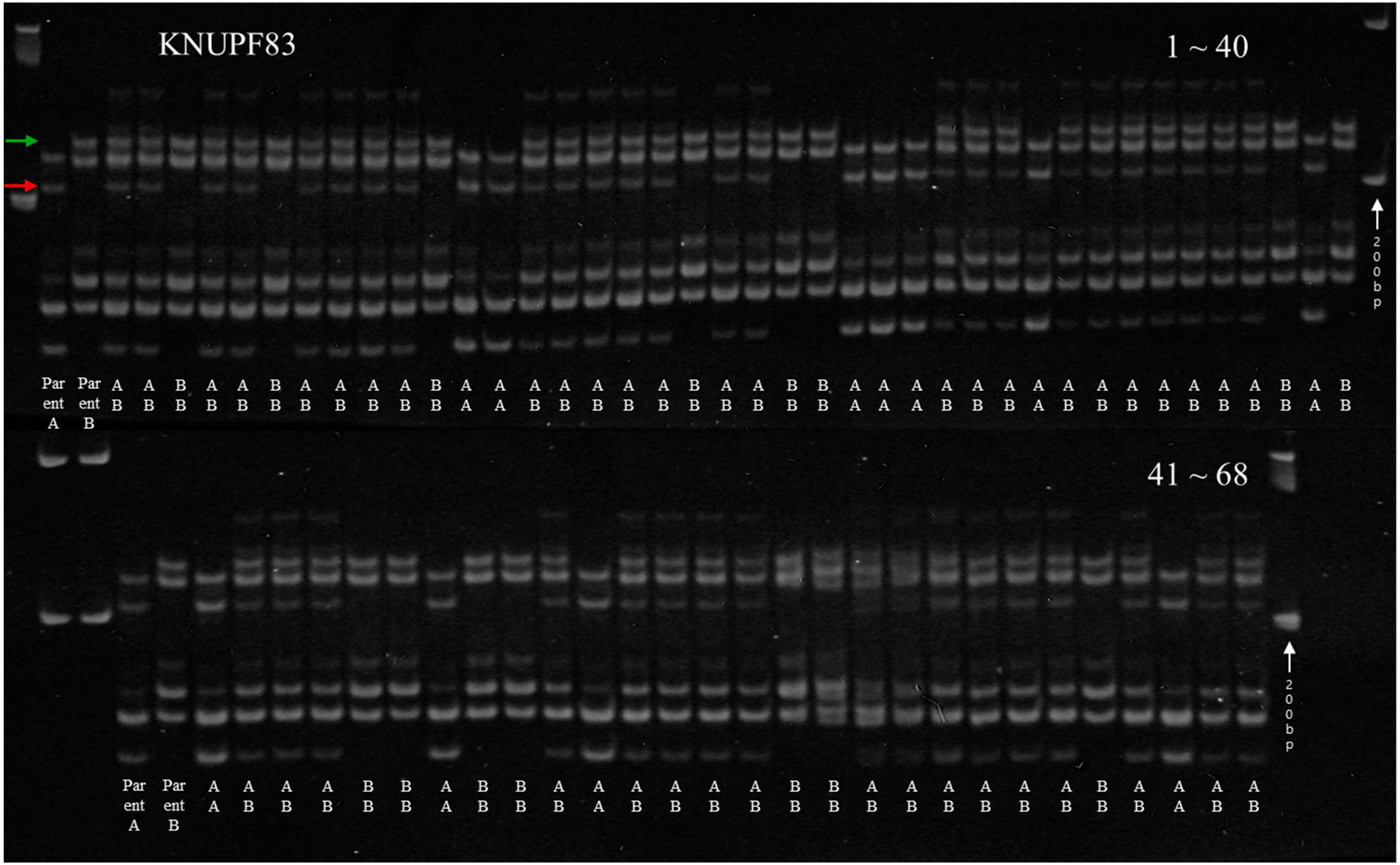

Supplementary Figure S1. Genotyping pattern of SSR marker KNUPF83 in 68 F_2 individuals and the two parental lines of *Perilla frutescens*.
