# Supplementary material for: Association Mapping Analysis of Morphological Characteristics in F2 Population of Perilla (Perilla frutescens L.) Using SSR Markers
Source: Plants (Basel). 2025 Sep 6;14(17):2799. doi: 10.3390/plants14172799 (PMC12430519; doi:10.3390/plants14172799)
Supplement: Supplementary file 1 [file plants-14-02799-s001.zip › Supplementary Table S1.pdf]

Supplementary Table S1. List of SSR markers used in this study with their sequence information and repeat motifs.

Marker	Forward Primer	Reverse Primer	Repeat motif
GBPFM179	TGAATCATCCCAAACGAGAT	TCGCTTCTCTCTCATGGATT	$(TGA)_5$
KWPE19	CAACCCTTCACGATCACTAT	AAATAACGGCCGATTCTAC	$(ACG)_7$
KWPE58	AGAGAGTTACCTGCGATTTTC	CTTCAATATTCGGCCATCTT	$[(TG)_9(AG)_{12}]$
KNUPF2	GAAACCAAATTTCTTGTTCTTACA	CAAACGCAGACTCTTATCAATG	$(AG)_{16}$
KNUPF3	TTCCTTGTAGTCATCTGATCCC	TGGAAATTAATTAAAGGGCTGA	$(AG)_{16}$
KNUPF4	TTTCAAAAATCTTACCAACGCT	TTCGTTTTTGCATCTAATTATTCA	$(AT)_{10}$
KNUPF9	ATGATGAATTGTGATGTGATGC	TACTACAAGGCAGGATTTTCGT	$(AT)_{11}$
KNUPF12	AATTCAATCTCGCCTCCATATT	TTCTGAATCTTGAAGCTTTGGT	$(CA)_{11}$
KNUPF14	AAATTCTCCCTCCACTCTTCAC	TGTTGGCTTTTTCAAATCTTTT	$(CT)_{12}$
KNUPF15	CCACACGTAAACCTCATAAACC	TTATCTCTAAAGAAATCGGGCA	$(CT)_{16}$
KNUPF16	CCTGTATCTCTCCCCGATAAAT	TGGATTTAATGCAGTTGAGTTG	$(CT)_{22}$
KNUPF23	TTGCAAGTTCTTGAATTGTGAC	CACTCCTTCCCTCCTCTTTAAT	$(TG)_{11}$
KNUPF29	CTGGAAGTTTCAGAGGAAAATG	GTCTAATCCGAACGAGAATCTG	$(AGT)_{10}$
KNUPF30	AACTAGTATATATGGCCTGCAAAAA	GACCTCTATCTCCCACATCCTA	$(ATC)_{10}$
KNUPF31	TAGAAGTGGGGGATCTGGA	AACTCGACATCCATTTGTATCG	$(ATC)_8$
KNUPF36	GGGAGACGAGATAACACATGAT	TGCATACTCGATTGAAAGAAGA	$(GCT)_8$
KNUPF37	GGTGTGAAAAAGAGAGTGGAGA	TTGAATTGCCTGTTGATAGTGA	$(GGT)_{10}$
KNUPF39	TCACCTTCCCCTTCATTTATTA	AGGATCGAACAGAACAAACTGT	$(TCT)_{13}$
KNUPF40	TTATCAAAGTCATCCCAACTCC	AGTTTGTTAGGGACGACGACTA	$(TCT)_8$
KNUPF42	CGAATTCAATAGGGAAAAATGA	AGACTCAAATCATAGGAGTTTACGA	$(AT)_7$
KNUPF50	TCGTGAATGAGGGTGGTG	GCTGCTATTGGCATTTCTTATG	$(CT)_{17}$
KNUPF59	AATCTCGATGCCTAACAACAGT	TTCCTTGTAAATCCAGCTAAGG	$(CAG)_7$
KNUPF61	GGGATACCCAAATTTCTACCAT	TCATGAAAAATCCAAACATTCA	$(CAG)_7$
KNUPF81	TTAAGCAACCAATTGCAGGTA	GTTGTGCAAAATTTGGTGATTT	$(AAT)_7$
KNUPF82	AAACCAAGGAACTCGTCAACTA	CGCTTCGTCTTTATTGTGTGTA	$(AGA)_7$
KNUPF83	TTTGTCTTGATCTGCTTTGATG	CTACCTCGCAGAATCAAGCTAT	$(TG)_9$
KNUPF93	GAAAACTCACGCCACAAATTAC	TTCCCATTAAGCAATTTTCACT	$(AT)_9$
KNUPF112	AGTTGGAGTGGTTAAACTTGGA	CACGCACACTCCAATACTACAA	$(AG)_{17}$
KNUPF127	GACGACTTCTCAATTTACAGCC	TATTTCCTGTTTTGTTTGGCTT	$(ATAC)_4$
KNUPF130	TGGGAAAATATCTCATTCTATTCA	CTACTGTTCATATTGCTTAGCTGC	$(ATAG)_5$
KNUPF156	TGATTAATTTTTCTTGCTCGGT	GAATTCAAACAAGAATTGGGAA	$(CA)_8$
KNUPF162	TGCTTCTGTTAATGAAAGCGTA	TATTCTCTTGCACGTTGATGTC	$(CT)_7$
KNUPF163	GCTTGTCAATGTTTCCCATATT	TTCTTTTCGGCTTCAATCAGT	$(CT)_7$
KNUPF167	TTCAACAGCTACAAAAATGCAC	TTTTCATTCACCGTTCACAAG	$(AG)_7$
KNUPF168	AAATTAATCCCAAAATAACGCC	GAAGCAGCAGTGAGAAATGAAT	$(AG)_8$
KNUPF169	ATTTCCTCCACACGCTTTTAAT	GCAACTCAGAACATCAGCACTA	$(CT)_{10}$
KNUPF170	ACTCGAACCCTCACTTTTGAC	ATTTCCGGAACCCTAAACTAAA	$(CT)_{10}$
KNUPF176	GACAAATTGGGAATACAGGAAA	TTCCGATTCAAGAAAGGTAGAA	$(CT)_{10}$
KNUPF182	TTGCCAAATTTTTACCATTATGT	CTACTTTGAGGCAAGGGTTTC	$(AG)_9$
KNUPF191	CGAGTCTCCGATACAACCAATA	GTTGAGTTTGAGCACACTGGAG	$(CT)_8$
