# Supplementary material for: Association Mapping Analysis of Morphological Characteristics in F2 Population of Perilla (Perilla frutescens L.) Using SSR Markers
Source: Plants (Basel). 2025 Sep 6;14(17):2799. doi: 10.3390/plants14172799 (PMC12430519; doi:10.3390/plants14172799)
Supplement: Supplementary file 1 [file plants-14-02799-s001.zip › Supplementary Table S2.pdf]

Supplementary Table S2. Morphological traits and their measurements in Parent A (var. *crispa*) and Parent B (var. *frutescens*).

Morphological trait	Unit or Category	Parent A	Parent B
QL1 (Color of leaf surface)	Green (1), Green/Purple (2), Purple (3)	Green/Purple	Green
QL2 (Color of leaf reverse side)	Green (1), Green/Purple (2), Purple (3)	Purple	Green
QL3 (Color of stem)	Green (1), Green/Purple (2), Purple (3)	Purple	Green
QL4 (Color of flower)	White (1), Pink (2), Purple (3)	Purple	White
QN1 (Days to heading)	day	124	119
QN2 (Days to flowering)	day	134	126
QN3 (Days to maturity)	day	168	157
QN4 (Plant height)	cm	151.4	105.4
QN5 (Length of inflorescence)	cm	8.6	8.5
QN6 (Number of florets)	Number	33.3	44
QN7 (Leaf length)	cm	12.6	12.6
QN8 (Leaf width)	cm	8.3	11.2
QN9 (Leaf area)	cm^2	59.8	88.8

Parent A: weedy type of *Perilla frutescens* var. *crispa*; Parent B: weedy type of *Perilla frutescens* var. *frutescens*; QL: Qualitative trait; QN: Quantitative trait

*Data previously published by Heo et al. (2025) [36]
