# Supplementary material for: Association Mapping Analysis of Morphological Characteristics in F2 Population of Perilla (Perilla frutescens L.) Using SSR Markers
Source: Plants (Basel). 2025 Sep 6;14(17):2799. doi: 10.3390/plants14172799 (PMC12430519; doi:10.3390/plants14172799)
Supplement: Supplementary file 1 [file plants-14-02799-s001.zip › Supplementary Table S3.pdf]

Supplementary Table S3. Pearson correlation coefficients among 13 morphological traits observed in the F_2 population of *Perilla frutescens*.

	QL1	QL2	QL3	QL4	QN4	QN3	QN1	QN2	QN5	QN6	QN7	QN8
QL2	0.353**											
QL3	0.584**	0.560**										
QL4	0.498**	0.625**	0.627**									
QN4	0.059	0.172	0.211	0.217								
QN3	0.145	0.044	0.188	0.236*	0.348**							
QN1	0.136	0.095	0.189	0.314**	0.279*	0.654**						
QN2	0.122	0.026	0.191	0.270*	0.319**	0.670**	0.779**					
QN5	-0.223	-0.016	-0.147	-0.290*	0.252*	0.027	-0.064	-0.02				
QN6	-0.187	-0.109	-0.268*	-0.314**	-0.022	-0.065	-0.119	-0.044	0.811**			
QN7	0.092	0.003	-0.095	0.002	0.146	0.092	0.091	0.123	-0.064	-0.027		
QN8	-0.085	-0.223	-0.396**	-0.352**	-0.255*	-0.192	-0.127	-0.141	-0.028	0.140	0.721**	
QN9	-0.067	-0.176	-0.353**	-0.296*	-0.091	-0.061	0.009	-0.008	-0.032	0.099	0.821**	0.921**

**Significance at $P < 0.01$; *Significance at $P < 0.05$.

QL1: Color of leaf surface; QL2: Color of leaf reverse side; QL3: Color of stem; QL4: Color of flower; QN1: Days to heading; QN2: Days to flowering; QN3: Days to maturity; QN4: Plant height; QN5: Length of inflorescence; QN6: Number of florets; QN7: Leaf length; QN8: Leaf width; QN9: Leaf area
